# Social support, stress coping strategies, resilience and posttraumatic growth in a Polish sample of HIV-infected individuals: results of a 1 year longitudinal study

**DOI:** 10.1007/s10865-017-9861-z

**Published:** 2017-05-31

**Authors:** Marcin Rzeszutek, Włodzimierz Oniszczenko, Ewa Firląg-Burkacka

**Affiliations:** 1 Faculty of Psychology, University of Warsaw, Stawki 5/7, 00-183 Warsaw, Poland; 2 Warsaw’s Hospital of Infectious Diseases, Wolska 37, 01-201 Warsaw, Poland

**Keywords:** HIV, Posttraumatic growth, Personal, Social, Longitudinal study

## Abstract

This study investigated the level of posttraumatic growth (PTG) and its association with the level of social support, stress coping strategies and resilience among a people living with HIV (PLWH) in a 1 year longitudinal study. We also controlled for age, HIV infection duration and the presence of posttraumatic stress symptoms (PTSS). From the 290 participants, initially eligible for the study, 110 patients were recruited for the first assessment and 73 patients participated in a follow-up assessment. Participants filled out following psychometric tools: the Posttraumatic Growth Inventory (PTGI), the Berlin Social Support Scales (BSSS), the Mini-COPE Inventory, the Resiliency Assessment Scale (SPP-25) and the PTSD-F questionnaire. Received support and resilience were positively, whereas return to religion as coping strategy was negatively related to the PTG. Clinicians and researchers need to focus on potentially positive consequences of HIV infection, i.e. PTG, and factors that might promote it among PLWH.

## Introduction

### Posttraumatic growth and HIV

Although decades of research have found negative consequences of HIV/AIDS (e.g. Israelski et al., 2007; King, 1993; Leserman, 2008; Safren et al., 2003; Rzeszutek et al., 2012, 2015), other studies have highlighted also the positive consequences of HIV infection, i.e. the occurrence of posttraumatic growth (PTG) (e.g. Milam, 2004, 2006; Murphy & Hevey, 2013; Sherr et al., 2011). There are several terms used to describe positive changes following traumatic events, such as benefit finding (Danoff-Burg and Revenson, 2005), stress-related growth (Siegel et al., 2005), thriving/flourishing (Sirois and Hirsch, 2013) or adversarial growth (McBride et al., 2009). In this study, however, we focused on posttraumatic growth’s definition by Tedeschi and Callhoun (1996, 2004), according to which PTG is defined as the set of positive changes in relations with others, self-perception and existential beliefs, in the form of greater appreciation of life and openness to spirituality, which can result from attempts at dealing with a traumatic or highly stressful life event. Particularly, people after these kinds of adverse life events may establish more satisfying relations with other people, start to recognize their strength in achieving new life goals and change their basic life values, which manifests by a shattering of their prior worldview.

Regarding people living with HIV (PLWH) and PTG, Milam (2004) in a longitudinal study (n1 = 835; n2 = 435) observed that 59% of HIV infected individuals reported some positive changes in the form of particular PTG dimensions, and these positive changes were negatively related to the level of depression and the intensity of substance use. Other advantages associated with PTG among PLWH were also found, including increased adherence to treatment and improvement of the immune system (Milam, 2006), as well as better psychological well-being and lower level of hospitalizations (Siegel et al., 2005). However, the majority of previous studies on PTG in PLWH were conducted in a cross-sectional framework and concentrated mainly on documenting particular PTG dimensions in this patient group and relating them to sociodemographic data, health status or HIV-related stigma (Murphy & Hevey, 2013; Sherr et al., 2011). Thus, knowledge about the psychological factors that might promote or hinder PTG in this sample is relatively scarce. In this study, we investigated the intensity of PTG and its association with the level of social support, stress coping strategies and resilience among a sample of PLWH in a 1 year longitudinal study.

### Social support and posttraumatic growth

According to Tedeschi and Calhoun (2004), social support received from close family and friends etc. (see, “supportive others”, Tedeschi & Calhoun, 2004, p. 8) helps people after traumatic events to express negative emotions and fosters cognitive processing (i.e. it mobilizes ruminative activity regarding the trauma, which is crucial in facilitating PTG). The degree of perceived support and the need for support, displayed in the intensity of support seeking, can also facilitate the use of more adaptive stress coping strategies (Tedeschi & Calhoun, 2004). Nevertheless, studies on the link between social support and PTG, especially in the aftermath of chronic illness, are inconclusive. Several authors have observed that social support may enhance PTG among cancer patients (Karanci & Erkam, 2007), rheumatoid arthritis patients (Dirik & Karanci, 2008) or stem cell transplant survivors (Nenova, 2013). However, Sheik (2004) found no association between social support and PTG among cardiac patients. In regard to PLWH, Cieślak et al. (2009) studied HIV infected survivors of Hurricane Katrina and found that received social support was positively linked only to one PTG subscale: relating to others. In addition, Wei et al. (2016) observed that perceived support mediated the link between stigma and PTG among children affected by the HIV/AIDS of their parents. Nevertheless, there is no consensus about the causal role of social support in PTG, especially among PLWH.

### Stress coping and posttraumatic growth

The way the individual copes with the traumatic event is very important to triggering PTG (Tedeschi & Calhoun, 2004). The most common stress coping strategies that are important for PTG are meaning-focused coping strategies, especially positive reappraisal (also referred to as positive re-evaluation, see Measures), which has been shown to be a significant PTG predictor among cancer patients (Sears et al., 2003) and HIV/AIDS population (Siegel & Shrimshaw, 2005). This kind of coping means making sense of one’s life after trauma and integrating it with existing cognitive schemas about the self and the world. One of the meaning-focused coping strategies that are important for PTG is religious coping. In fact, according to a meta-analysis conducted by Prati and Pietrantoni (2009), of the many stress coping strategies, positive reappraisal and religious coping have the largest effect on PTG. Conversely, avoidance coping strategies, such as substance use hinder the probability of growth after trauma (Helgeson et al., 2006). This latter stress coping strategy is frequently used among PLWH to reduce HIV-related distress and may be related to perceived dissatisfaction with social support and low treatment adherence (Power et al., 2004). It is also worth mentioning that the effectiveness of stress coping depends not only on the characteristics of the traumatic event but also on the personal traits of the individual and the social environment (Morris et al., 2005).

### Resilience and posttraumatic growth

The term resilience may be defined either as a process of successful adaptation to trauma and adversity (Bonano, 2004) or a personality trait, which refers to the degree of emotional stability after experiencing very stressful of traumatic events (Block & Kremen, 1996). In this study we concentrated on resilience as a personality trait. The link between resilience and PTG is ambiguous. Some authors observed that resilience is positively related to PTG (Bensimon, 2012; Westphal and Bonanno, 2007). Conversely, Tedeschi and Calhoun (2004) underlined that aforementioned variables may be negatively associated, as resilience acts only as a buffer that protects an individual from the negative consequences of trauma and adversity, but it does not promote PTG. In other word, whereas a resilient person usually recovers from a traumatic event without psychological disturbances, PTG means unexpected transformation, displayed in a level of functioning that is higher than before the trauma. Regarding PLWH, only Murphy and Hevey (2013) have investigated the role of resilience in PTG among this patient group, finding a positive link between these two variables in their cross-sectional study.

## Conceptual framework

This study investigated the level of PTG, as the explained variable, and its association with the levels of social support dimensions, the intensity of stress coping strategies and the level of resilience, defined as a personality trait, among a sample of HIV- infected patients in a one-year longitudinal study. Age, HIV infection duration and the presence of posttraumatic stress symptoms (PTSS) were also controlled in the study sample. Three hypotheses were formulated according to the longitudinal study framework (Cole & Maxwell, 2003):

Social support

1. It was expected a positive relationship between the levels of received support in the first assessment and the intensity of PTG in the follow-up assessment, while controlling for the level of PTG in the first assessment. In addition, it was expected that perceived support and need for support will mediate the link between received support, stress coping and the global PTG score.

Coping mechanisms

2. It was expected a positive relationship between meaning-focused coping strategies (positive re-evaluation, return to religion), and a negative relationship between avoidance coping strategies (substance use) in the first assessment and the intensity of PTG in the follow-up assessment, while controlling for the level of PTG in the first assessment.

Resilience

3. It was expected a positive association between the intensity of resilience in the first assessment and the intensity of PTG in the follow-up assessment, while controlling for the level of PTG in the first assessment.

**Fig. 1:**
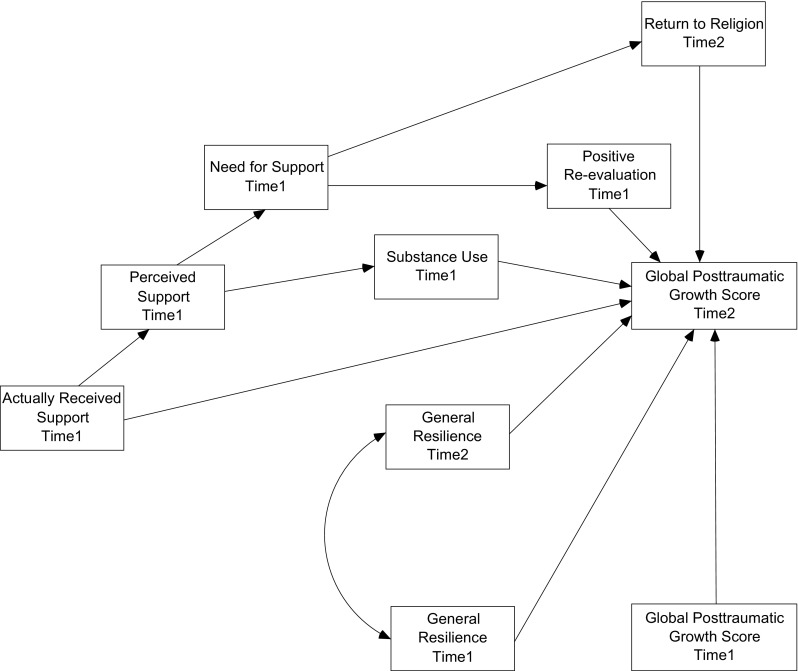
Hypothethised Path Diagram of the Relationship Between Social Support Dimensions, Stress Coping Strategies, Resilience as a Trait and the Intensity of the Global Posttraumatic Growth Score in HIV + Patients (*n* = 73)

A preliminary model was created, in which we expected a positive relationship between received support, resilience measured in the first and the follow-up assessment, return to religion and positive re-evaluation as meaning-focused coping strategies and the global PTG score (explained variable). Conversely, a negative relationship between substance use as an avoidance coping strategy and the global PTG score was expected. It was also hypothesised that perceived social support and need for support would act as mediators between received support, stress coping strategies (return to religion, positive re-evaluation, substance use) and the global PTG score. It was also expected that perceived social support would mediate the relationship between received support and substance use. The hypothesised model is depicted in Fig. 1.

## Method

### Participants and procedure

After the informed consent was obtained, the participants completed a paper-and-pencil version of the inventories and participated in the study voluntary, as there was no remuneration for the participation. The research questionnaires were distributed by the patients themselves in paper form by the authors of this study and professional interviewers to the patients in the Hospital of Infectious Diseases in Warsaw. The eligibility criteria were 18 years of age or older, a confirmed medical diagnosis of HIV infected and receiving care from the hospital where the study was conducted. The exclusion criteria encompassed HIV-related cognitive disorders, which were screened by psychiatrists working in the hospital, where the study was conducted. The research project was accepted by the Senate Ethics Committee of the University of Finance and Management in Warsaw.

The first assessment was conducted between June and July 2015 in the Hospital of Infectious Diseases in Warsaw. From the 290 patients with a clinical diagnosis of HIV infection, eligible for the study, 110 patients (38%) were recruited for the first assessment, i.e. those patients agreed not only to fill the questionnaires, but also agreed to leave their contact details (i.e. telephone number and/or e-mail address) so the authors of this study could contact them for a one-year follow-up, and additionally indicated in the Posttraumatic Growth Inventory (see, Measures) that the diagnosis of HIV infection was a traumatic event for them. 140 patients (48%) refused to leave their contact details, 22 patients (8%) refused to fill the questionnaires and 18 patients (6%) completed the questionnaires with a level of missing data exceeding 50% of the study questionnaires, which precluded including them into the statistical analysis. Specifically, in the first assessment there were 105 men and 5 women from 19 to 76 years of age (*M* = 39.45; *SD* = 11.86). The duration of HIV infection among patients in the baseline assessment varied between 1 and 28 years (*M* = 7.19; *SD* = 6.99). The second assessment was conducted between June and July 2016, and out of the 110 patients who left contact details 1 year earlier, 73 patients agreed to participate in the second assessment; further statistical analysis was conducted on this sample. There were 68 men and 5 women from 19 to 76 years of age (*M* = 38.77; *SD* = 12.61). The duration of HIV infection among patients in the second assessment varied between 1 and 28 years (*M* = 6.42; *SD* = 6.63). The total response rate was 66% (73/110). There was no missing data in the final sample of 73 participants. There was no missing observations estimation method applied.

Participants who refused to take part in the follow-up assessment did not differ from the sample that agreed to take part in the follow-up study with respect to age (*t* (108) = −.85, *p* > .05) or HIV infection duration (*t* (108) = −1.63, *p* > .05). However, the individuals who refused to participate in the follow-up assessment had significantly higher levels of global PTSS than those who agreed to participate in the follow-up study (*t* (108) = −8.09, *p* < .001).

### Measures

To measure the intensity of posttraumatic growth, the Posttraumatic Growth Inventory was used (PTGI; Tedeschi & Calhoun, 1996) in a Polish adaptation by Ogińska-Bulik and Juczyński (2010). It is important to underline that although the original PTGI comprises 5 specific domains of PTG (“relating to others”, “new possibilities”, “personal strength”, “spiritual change, and appreciation of life”), the Polish adaptation of PTGI measures only four domains of posttraumatic growth. Exploratory and confirmatory factor analysis revealed a four-factor structure for the PTG, including changes of perception of oneself (“perceiving new possibilities, feeling of personal strength”), changes in relationships with others (“feelings of greater connection with other people, increase in empathy, altruism”), greater appreciation for life (“changes in life philosophy and current life goals, greater appreciation for every day”), and spiritual changes (“better understanding of spiritual issues, increase in religiousness”) (Ogińska-Bulik & Juczyński, 2010). In the PTGI, participants have to rate 21 positive statements that describe various changes resulting from traumatic or highly stressful events, which are highlighted at the beginning of the inventory. Participants were instructed to focus on their HIV infection and as the example of traumatic experience. Global PTG score is obtained when one calculates all items of the inventory. The Cronbach’s α for the whole scale in the current study was .85 and for the four subscales varied between .83 and .85.

Social support was measured by the Berlin Social Support Scales (BSSS), in a Polish adaptation of Łuszczyńska et al. (2006). The BSSS are comprised of scales used to evaluate different dimensions of social support. The BSSS measures different components of social support and in this study following scales were used: perceived support (the extent to which help from others is available), need for support (the extent to which support in stressful situations is important to the participant), and received support (the real quantity of support received from others). The Cronbach’s *α* reliability coefficients for all scales in the current study were satisfactory as well, fluctuating between .84 and .85.

Stress coping strategies were evaluated by Carver’s Mini-COPE Inventory in the Polish adaptation of Ogińska-Bulik and Juczyński (2009). The Mini-COPE Inventory measures dispositional stress coping, defined as the typical pattern of reactions and feelings under high stress for a particular person. This inventory consists of 28 items, which form 14 subscales describing several stress coping strategies, including problem-focused coping (“active coping, planning, seeking instrumental support”), emotion-focused coping (“seeking emotional support”, “acceptance”, “sense of humour”), meaning-focused coping strategies (“positive re-evaluation, return to religion”) and avoidance coping strategies (“self-distraction, denial, venting, substance use, behavioral disengagement, and self-blame”). Cronbach’s *α* for the Mini-COPE in the current ranged from .79 to .87.

The level of resilience as a personality trait was assessed with the Resiliency Assessment Scale (SPP-25), constructed by Ogińska-Bulik and Juczyński (2008). This scale consists of 25 items and provides a general resilience score and scores on five subscales describing particular aspects of resilience: “persistence and determination in action”; “openness to new experiences and a sense of humour”; “personal skills to cope and tolerance of negative emotions”; “tolerance of failure and viewing life as a challenge”; “an optimistic attitude towards life and the ability to mobilize in difficult situations”. Respondents rate the answers on a 5-point Likert-type scale. Cronbach’s *α* for the whole scale in the current study was .84, and for the five subscales varies between .84 and .85.

To measure the level of posttraumatic stress symptoms as a control variable in the studied patient group, the PTSD Factorial Inventory (PTSD-F; Strelau et al., 2002) was used. This inventory contains 30 items, which are divided into three scales: intrusion/arousal (recurrent thoughts relating to the traumatic event and causing arousal; 15 items), avoidance/numbing (avoidance of trauma-related stimuli and weakened response to these stimuli; 15 items), and a global trauma score (all 30 items). Patients are asked to report how often in the past several months they experienced a given thought, behaviour, or emotion related to the traumatic event—participants were instructed to focus on their HIV infection. The PTSD-F has satisfactory psychometric properties for the current study: assessed with Cronbach’s α, the reliabilities for the intrusion/arousal scale, the avoidance/numbing scale, and the global trauma score were .85, .85, and .86, respectively.

### Data analysis

Analytic plan consisted of three stages. Each variable was measured twice. Firstly, possible differences between two assessments were examined. *T* test for dependent variables was used when distribution of the variables did not deviate from the normal distribution and Wilcoxon signed-rank test was employed when distribution of the variables differed from the normal distribution. Because the sample size was relatively small we did not apply correction for multiple comparisons, i.e. Bonferroni correction, but computed Cohen’s *d* effect size for each comparison. The use of Bonferroni correction is criticized for loss of statistical power, which is especially important when the sample size is small. In this case, reporting of effect size measures is recommended (see, Nakagawa, 2004) and we followed those guidelines.

Second stage of the analysis was devoted to the selection of control variables. It was assumed that besides the post-traumatic growth level in the first assessment, participants’ age, HIV infection duration and the global posttraumatic stress symptoms score should be controlled for as well, but only if they are significantly related to the explained variable, which was the post-traumatic growth level in the second assessment. Multiple regression analysis with the use of the entry method was applied to make an appropriate decision (Kutner et al., 2004).

The final stage of the analysis was concerned with the posttraumatic growth process. Hypothetical model explaining which variables and in what order led to the increase in post-traumatic growth level in the second assessment with respect to the first assessment was created. The model contained associations between variables that were mentioned in the three formulated hypotheses: relationship between the levels of received support and the intensity of PTG (hypothesis 1.), relationship between meaning-focused coping strategies, avoidance coping strategies and the intensity of PTG (hypothesis 2.) and association between the intensity of resilience and the intensity of PTG (hypothesis 3.). Model, in which received support leads to the global PTG score in the follow-up assessment among participants, while controlling for the level of the global PTG score in the first assessment was verified. The global PTG score in the follow-up assessment was defined as an explained variable, and received support, measured the first time, was defined as an exogenous variable. Path analysis based on the maximum likelihood method was applied to verify and modify the assumed model. Appropriate modifications were made on the basis of modification indices with the threshold level of 4. IBM SPSS 24 and IBM AMOS 24 statistical package was used to conduct the statistical analysis (IBM Corp. Released, 2016).

## Results

### Differences between the two assessments

First, for descriptive reasons, means and standard deviations of two assessments with respect to variables in the PTGI, BSSS, Mini-COPE, SPP-25 and PTSD-F were calculated, using a *t* test for dependent samples and a Wilcoxon signed-rank test when the normal distribution was not achieved.

As can be seen in Table 1, a significant increase in the levels of particular PTG dimensions (see, changes of perception of oneself, greater appreciation for life) as well as in the g*lobal PTG score* among the participants were observed. Achieved effect sizes ranged from Cohen’s *d* = −.22 to Cohen’s *d* = −.26. In addition, a significant increase in the intensity of return to religion (*d* = −.55) and behavioral disengagement (*d* = −.27), and a significant decrease in the level of active coping (*d* = .29) and positive re-evaluation (*d* = .25) as a stress coping strategy were observed. Finally, no differences in the levels of social support dimensions, resilience and PTSS among the participants between the two assessments were observed. Further analyses were performed only for the global PTG score, the general resilience score and the global PTSS score, as particular subscales in the Polish version of the PTGI inventory, the SPP-25 questionnaire and the PTSD-F questionnaire are highly intercorrelated (see, Ogińska-Bulik & Juczyński, 2008, 2010; Strelau et al., 2002).

**Table 1:** Means and standard deviations comparisons for two assessments of the variables in PTGI, the BSSS, the MINI-Cope, SPP-25 and the PTSD-F inventory in HIV + Sample (*n* = 73)

Variables	Time 1 M (SD)	Time 2 M (SD)	Dependent-sample *t* test value	Cohen’s *d*
PTGI				
Changes of perception of oneself	22.27 (12.30)	25.15 (10.56)	−2.15*	−.25
Changes in relations with others	16.89 (9.12)	19.07 (8.73)	−1.54(a)	−.22
Greater appreciation for life	8.45 (4.50)	9.53 (3.58)	−.2.07*	−.24
Spiritual changes	3.29 (2.82)	3.74 (2.96)	−1.17(a)	−.14
Global posttraumatic growth score	50.90 (25.92)	57.49 (22.60)	−2.26*	−.26
BSSS				
Perceived support	18.59 (5.50)	19.10 (4.60)	−.06(a)	−.08
Need for support	7.12 (2.83)	7.52 (2.24)	−.95(a)	−.14
Actually received support	32.73 (10.39)	31.02 (9.29)	−1.36(a)	.13
MINI-COPE				
Active coping	4.52 (1.29)	3.99 (1.59)	−2.35*(a)	.29
Planning	4.37 (1.25)	4.04 (1.53)	−1.30(a)	.18
Seeking instrumental support	3.67 (1.76)	3.96 (1.42)	−1.08(a)	.08
Seeking emotional support	3.67 (1.76)	3.52 (1.84)	−.57(a)	−.15
Positive re-evaluation	4.05 (1.35)	3.60 (1.43)	−2.09*(a)	.25
Acceptance	4.51 (1.30)	4.25 (1.37)	−1.26(a)	.14
Sense of humour	2.76 (1.31)	2.95 (1.44)	−.90(a)	−.10
Return to religion	1.68 (1.99)	2.93 (1.98)	−4.30***(a)	−.55
Self-distraction	3.38 (1.61)	3.33 (1.44)	−.46(a)	.03
Denial	1.84 (1.77)	2.03 (1.72)	−.70(a)	−.09
Venting	3.15 (1.42)	3.23 (1.38)	−.13(a)	−.05
Substance use	1.71 (1.90)	2.22 (2.07)	−1.53(a)	−.20
Behavioral disengagement	1.68 (1.53)	2.19 (1.41)	−2.54*(a)	−.27
Self-blame	3.14 (1.59)	3.25 (1.51)	−.40(a)	−.06
SPP-25 scales				
Persistence and determination in action	14.04 (3.76)	14.16 (3.70)	−.26	−.03
Openness to new experiences and a sense of humour	16.11 (3.36)	15.40 (3.56)	−1.63(a)	.17
Personal skills to cope and tolerance of negative emotions	14.12 (3.93)	14.55 (3.92)	−1.29(a)	−.09
Tolerance of failure and view life as a challenge	14.74 (3.65)	14.79 (3.70)	−.49(a)	−.01
Optimistic attitude towards life and the ability to mobilize in difficult situations	13.39 (3.84)	14.04 (4.15)	−1.23	−.14
General resilience score	72.41 (16.53)	72.95 (17.66)	−.24	−.03
PTSD-F				
Intrusion/arousal	14.45 (10.31)	13.43 (9.21)	−.47(a)	.08
Avoidance/numbing	12.63 (10.23)	11.96 (9.15)	−.24(a)	.06
Global posttraumatic stress score	27.08 (19.27)	25.40 (17.81)	−.39(a)	.07

*Note* Time 1—first assessment; Time 2—follow-up assessment; (a) Z = value for Wilcoxon signed-rank test; * *p* < .05; *** *p* < .001

### Selection of control variables

Second, to examine whether the control variables (i.e. age, HIV infection duration and the global posttraumatic stress symptoms score) in the first assessment might be related to the global PTG score in the follow-up assessment (the explained variable), while controlling for the level of the global PTG score in the first assessment among participants, a multiple regression analysis via the entry method has been conducted (Kutner et al., 2004). In each step, the statistical significance of the increment in the explained variance was measured based on the *F*-change indicator, and the final model presents a semi-partial correlation for each independent variable. The results are presented in Table 2.

**Table 2:** Model coefficients for regression analysis predicting global PTG score in the follow-up assessment (explained variable), while controlling for the PTG score in the first assessment, in respect to age, HIV infection duration and global posttraumatic stress symptoms in the first assessment among HIV + Participants (*n* = 73)

Model	*F*	*F* Δ	*R*	*R* ^2^	Predictor	Semi-partial correlation
Global posttraumatic growth Score (T1)	21.12(a)***	–	.48	.23	Global posttraumatic growth Score (T1)	.48***
Global posttraumatic growth Score (T1)	10.70(b)***	.45	.48	.23	Global posttraumatic growth Score (T1)	.47***
+ Age (T1)					Age (T1)	−.07
Global posttraumatic growth Score (T1)	7.06(c)***	.08	.48	.24	Global posttraumatic growth Score (T1)	.47***
Age (T1)					Age (T1)	−.05
+ HIV infection duration (T1)					HIV infection duration (T1)	−.03
Global posttraumatic growth	5.85(d)***	1.92	.51	.26	Global posttraumatic growth	.48***
Score (T1)					Score (T1)	
Age (T1)					Age (T1)	−.05
HIV infection duration (T1)					HIV infection duration (T1)	−.02
+ Global posttraumatic stress					Global posttraumatic stress symptoms	−.15
Symptoms score (T1)					Score (T1)	

*Note* Explained variable: Global Posttraumatic Growth Score in the Follow-up- Assessment (T2); T1—First Assessment

(a) *df* = 1,71, *** *p* < .001

(b) *df* = 2,70, *** *p* < .001

(c) *df* = 3,69, *** *p* < .001

(d) *df* = 4,68, *** *p* < .001

As can been seen in Table 2, age, HIV infection duration and global posttraumatic stress symptoms score were not related to the explained variable, so these variables were not include in the next part of statistical analysis.

### Model of posttraumatic growth process

The last step of the statistical analysis was devoted to the creation of a path model. The AMOS graphics program was used to create a path diagram displaying process, in which received support leads to the global PTG score in the follow-up assessment among participants, while controlling for the level of the global PTG score in the first assessment. This study was based on the maximum likelihood method. The global PTG score in the follow-up assessment was defined as an explained variable, and received support, measured the first time, was defined as an exogenous variable. Preliminary, a model was created, in which a positive relationship was expected between received support, resilience measured in the first and follow-up assessments, return to religion and positive re-evaluation—as meaning-focused stress coping strategies—and the global PTG score (explained variable). Conversely, a negative relationship between substance use as an avoidance coping strategy and the global PTG score was expected. It was also hypothesised that perceived social support and need for support would act as mediators between received support, stress coping strategies (return to religion, positive re-evaluation, substance use) and the global PTG score. It was also thought that perceived social support would mediate the relationship between received support and substance use. The hypothesised model was depicted in Fig. 1 in the conceptual framework section.

**Fig. 2:**
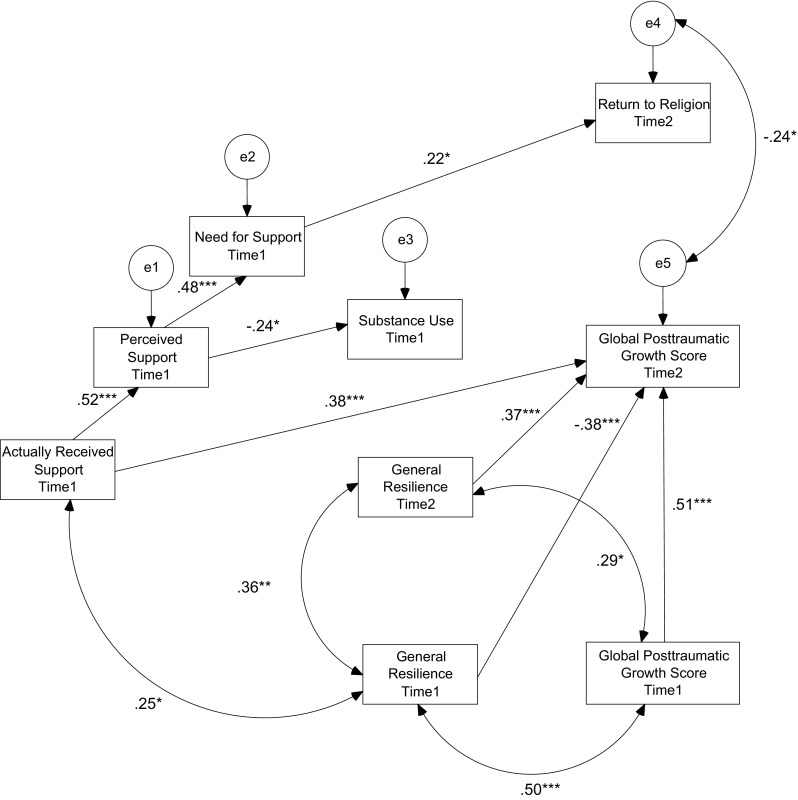
Final path diagram of the relationship between social support dimensions, stress coping strategies, resilience as a trait and the intensity of the global posttraumatic growth score in HIV + Patients (*n* = 73). *p < .05; **p < .01; ***p < .001

Contrary to preliminary expectations, the following paths were not significant: between substance use and the global PTG score (explained variable), between positive re-evaluation and the global PTG score (explained variable) and between positive re-evaluation and return to religion. Therefore, these paths were removed from the model, and in order to additionally increase the model fit, the regression path between return to religion and the global PTG score was changed into a covariance. A covariance between received support and resilience from the first assessment was also added. The final model is presented in Fig. 2.

The model contains relations between explanatory variables. Effect size was interpreted following Cohen’s guidelines (1988). The magnitude of positive correlation between *general* resilience and received support was small. The magnitude of positive correlation between general resilience and global posttraumatic growth score was high. Received support explained 27.2% of perceived support’s variance (strong effect size). Perceived support explained 23.4% (medium effect size) of need for support’s variance and 6.0% of substance use’s variance (weak effect size). The model contains three tested predictors of the explained variable (received support and general resilience from the first and second assessment) and one controlled variable (the global posttraumatic growth score in the first assessment). Achieved statistical power was .78. Finally, the values of goodness of fit indices suggested that the model fit was very good (*chi* ^*2*^ = 23.14; *df* = 23; *p* > .05; *CFI* = .999; *RMSEA* = .009).

It was found that received support was positively related to the global PTG score in the follow-up assessment (*beta* = .38) as well as to the level of resilience in the first assessment (*r* = .25). It was also observed that perceived support and need for support mediated only the link between received support and stress coping strategies (return to religion). It was also found that perceived support mediated the relationship between received support and substance use, i.e. perceived support led to lower level of substance use. Finally, resilience, in the first assessment, was positively related to the global PTG score in the first assessment (*r* = .50). Similarly, resilience in the second assessment was also positively related to PTG in the follow-up assessment (*beta* = .37). However, resilience in the first assessment was negatively related to the global PTG score in the second assessment (*beta* = −.38), which means that participants with a higher level of resilience in the first assessment experienced a lower increase in PTG between the two assessments. Comparing the hypothetical model with the final model we would like to highlight that positive re-evaluation was supposed to be important coping strategy for PTG, but the results did not confirm its role. We did not find evidence that substance use inhibits PTG. It was supposed that return to religion leads to PTG, but it was found that these two variables correlated negatively with each other. PTG in the first assessment correlated with general resilience in the first and in the second assessment.

## Discussion

The first hypothesis was supported, as received social support was directly, positively related to the global PTG score at the one-year follow-up. Received social support has been shown to be a PTG-promoting factor in the HIV infected sample (Cieślak et al., 2009), as well as in many non-HIV samples (Nenova 2013). In particular, Cieślak et al. (2009) found that received social support was positively associated with one PTG subscale (i.e. relating to others) and these authors argued that they examined the role of received support only 2 months prior to the study, so perhaps the significance of received support for the global PTG score appears over a longer period of time. Likewise, also in our study received social support was directly, positively associated with PTG score at the one-year follow-up. Finally, the model offered evidence that perceived support and need for support mediated the relationship between received support and stress coping. According to Tedeschi and Calhoun (2004), the degree of perceived support and the need for support, which are displayed in the intensity of support seeking, can facilitate the use of more adaptive stress coping strategies. In particular, Peterson et al. (2011) observed that social support may enhance various stress coping strategies, thus improving well-being among PLWH. Specifically, perceived support led to a lower level of substance use among participants, which corresponds with the findings of Rothman et al. (2008), who observed that drug use were related to dissatisfaction in perceived social support among PLWH.

The second hypothesis was not supported. On one hand, a negative link between return to religion as a meaning-focused coping strategy (covariance) was observed and, at the same time, no association between positive re-evaluation as a meaning-focused coping strategy and the global PTG score among was found the study sample. Additionally, there were no direct links between avoidance coping (substance use) and the global PTG score among participants. The negative relationship between return to religion and the global PTG score was an intriguing result and can be explained in two ways. First, some authors highlighted that in certain situations religious coping may not only be unrelated to PTG but also hinder growth after trauma, when a person lays the blame and responsibility for his or her disease on God (or some other force majeure), which strengthens passivity and contributes to giving up on medical treatment (Pargament, 2007). Wanyama et al. (2007) showed that religious beliefs about HIV may cause fatalistic attitudes and resignation from treatment. Furthermore, Zou et al. (2009) observed that moral connotations usually associated with HIV infection can turn the religious community into a stigmatizing atmosphere for PLWH, which can lead them to withdraw from such a community. Conversely, the aforementioned result may be understood to indicate that receiving support in PLWH may have two, separate, positive consequences: an increase in the level of PTG or an increase in the intensity of return to religion, mediated by perceived support and need for support (see Table 1; Fig. 2). Perhaps those PLWH who engage in religious coping may not experience growth while those who experience growth are not interested in searching for relief in religion. Nevertheless, this latter explanation requires further study. In addition, the lack of association between positive-re-evaluation and PTG among the study sample proved that this coping strategy may not necessarily be important for PTG promotion, especially taking into account that the level of positive re-evaluation decreased over 1 year among participants. Even so, this needs further investigation. Finally, the lack of a direct relationship between substance use as an avoidance coping strategy and the global PTG score can likely be attributed to perceived support, which was negatively related to this stress coping strategy among participants.

The last hypothesis was supported, as resilience as a personality trait, in the first assessment, was positively related to the global PTG score in the first assessment. Likewise, the level of resilience in the second assessment was also positively related to the global PTG score in the second assessment. Interestingly, resilience level in the first assessment was negatively related to the global PTG score in the second assessment, while the global PTG score in the first assessment was controlled for, which means that participants with a higher level of resilience in the first assessment experienced a smaller increase in PTG between the two assessments. Moreover, this is not due to a ceiling effect, because there was only one participant with the highest possible PTG score in the second assessment, which is 105 points.

This result corresponds with those of other studies indicating that resilience facilitates the probability of PTG in various populations after traumatic events (Bensimon, 2012). Murphy and Hevey (2013) showed that resilience was positively associated only with outcomes in the domains of personal strength and appreciation of life. According to Walsch (2007), resilient people are capable of rebounding from traumatic or highly stressful events and adapt to change due to changes in cognitive schemas, which are similar to those observed in PTG. In addition, resilience is closely related to other personality factors, which are positively related to PTG, such as sense of coherence, self-efficacy or optimism (Bensimon, 2012). The role of this latter variable (i.e. optimism in PTG promotion among PLWH) was proven by Milam (2006) in a longitudinal study.

No significant relationship between the level of PTSS and the global level of PTG was found among participants. Previous research has not reached a consensus on the link between PTG and PTSS. While Frazier et al. (2001) observed a negative relationship between PTG and PTSS. Conversely, Tedeschi and Calhoun (1996) found that higher level of PTSS is inevitable to facilitate growth after trauma. The positive link between PTG and PTSS was observed in HIV infected sample (Cieślak et al., 2009). In particular, Rzeszutek et al. (2016) in a cross-sectional study found a positive association between PTG and PTSS, but only among HIV infected women. In addition, Kleim and Ehlers (2009) wrote about curvilinear relationship between this constructs PTG and PTSD. Furthermore, there are studies highlighting the lack of a significant association between PTG and PTSS (see, Salsman et al., 2009), which was proven in this study.

There was also no significant relationship between the participants’ age and HIV infection duration and the global PTG score among participants. Studies on the link between age and PTG are equivocal. Some authors found a higher intensity of PTG among younger people (Helgeson et al., 2006). Conversely, other studies showed that older people, facing the imminence of death, can have a greater sense of meaning and openness to spiritual issues (Karanci & Erkam, 2007). Similarly, inconsistent findings can be found in the literature regarding the link between the amount of time since a traumatic event and PTG. Frazier et al. (2001) observed a negative correlation between PTG and time since a sexual assault. Conversely, Park and Fenster (2004) found that the longer the period after a cancer diagnosis, the higher the intensity of PTG. Furthermore, Prati and Pietrantoni (2009) in a meta-analysis, underlined that the time since trauma is not a significant moderator of the link between personal (optimism, stress coping) and social resources (social support) and PTG in many samples after trauma. The lack of an association between HIV infection duration and PTG in the study sample could indicate that HIV disease stage is not related to growth. Substantial progress in antiretroviral therapy has led to a decrease in HIV-related mortality in the last decade, and many authors now perceive HIV infection more as a chronic rather than terminal illness (Deeks et al., 2013). Siegel and Shrimshaw (2005) underlined that the most critical moment for PLWH is the moment of being diagnosed with HIV, which may result in modifications in the individual’s current beliefs and cognitive schemas, comprising the core elements of PTG. Nevertheless, participants had various lengths of HIV infection, which may also explain the lack of association between HIV infection duration and PTG in this study.

Finally, it is worth mentioning that this study may be important for Polish HIV infected individuals, as each year the number of new HIV infections in Poland increases by 13–14% (Supreme Audit Office, 2015). In addition, HIV education and prevention in Poland remain at a relatively poor level. Particularly, the majority of the funds from the National Programme for Preventing HIV Infections and Combating AIDS is spent on treatment, and not on prevention and education, which is responsible for that increasing number recently infected individuals in Poland do not know about their HIV-positive status. Furthermore, high levels of HIV-related stigma and discrimination may be still observed in Poland and the access to mental health care for HIV/AIDS population is rather scarce (Skonieczna, 2013). In the light of aforementioned factors, continuing research on psychological aspects of HIV/AIDS in Poland, including research on PTG is fully justified.

## Limitations

It is vital to mention the limitations of this study. There was only one follow-up assessment, and the follow-up cohort of participants was relatively low, so the size of the statistical effects is not very high (see Table 1). Perhaps more follow-up assessment s would answer the question of whether the relationships between the study variable are, for example, curvilinear. In addition, other social support scales have not been examined (e.g. those who provided social support) that may be associated with PTG in participants. In the study sample, there was also a significant underrepresentation of HIV infected women, so no gender differences could be found. Another limitation is that data were collected from a convenience sample that suffered from substantial loss to follow-up. In addition, as was stated previously, participants had various durations of HIV infection, which may have influenced the shape and magnitude of some relationships, such as the association between HIV infection duration and PTG or the lack of relationship between PTG and PTSD symptoms. Finally, demographic data have not been investigated thoroughly (e.g. education level, employment, religious affiliation, sexual orientation) or HIV-related variables (e.g. HIV transmission, treatment).

## Conclusions

Despite these limitations, the current study provides new insights into the personal and social predictors of posttraumatic growth in a sample of people living with HIV in Poland. Clinicians and researchers need to focus on potentially positive consequences of HIV infection, i.e. PTG. As PTG is related to several health-related benefits in this patient group (Milam, 2004, 2006) and the substantial amount of variation of HIV infection progression is still relatively poorly understood, further exploration of this topic is necessary.
